# In Silico Investigation on the Molecular Behavior and Structural Stability of the Rosette Nanotubes as the Drug Vehicles for Paclitaxel, an Anti-Cancer Drug

**DOI:** 10.3390/molecules28237853

**Published:** 2023-11-29

**Authors:** Hanah Nasifa M. Ali, Arthur A. Gonzales

**Affiliations:** Department of the Chemical Engineering, University of the Philippines Diliman, Quezon City 1101, Philippines; hmali@up.edu.ph

**Keywords:** rosette nanotube, paclitaxel, nano-based drug delivery system, molecular dynamics, MMPBSA

## Abstract

Most anticancer drugs affect healthy cells in addition to cancer cells, causing severe side effects. Targeted delivery by nano-based drug delivery systems (NDDS) can reduce these severe side effects while maintaining therapeutic efficacy. This work introduced rosette nanotube (RNT) as a potential drug vehicle for paclitaxel (PTX) due to its self-assembling property, biocompatibility, amphiphilicity, and low toxicity. Molecular dynamics (MD) simulations aided with molecular mechanics Poisson Boltzmann surface area (MMPBSA) analysis are used here to investigate the molecular behavior and the loading energetics of each type of RNT (K1, xK1, and iEt-xK1) with PTX. Analysis showed that the most probable configuration of PTX is on either end of each RNT. The binding free energies (−117.74 to −69.29 kJ/mol) when PTX is closer to one end were stronger than when it is in the inner channel (−53.51 to −40.88 kJ/mol). The latter alludes to the encapsulation of the PTX by each RNT. Thus, loading is possible by encapsulation during the self-assembly process given the favorable estimated binding free energies. Based on the results, RNT has potential as a drug vehicle for PTX, which warrants further investigation.

## 1. Introduction

**Figure 1:**
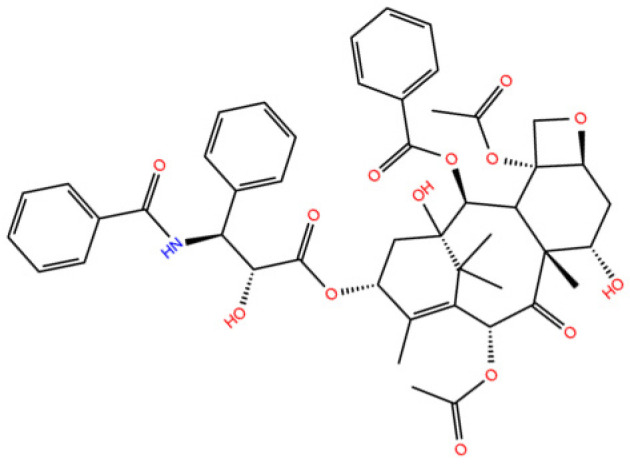
2D molecular structure of Paclitaxel.

Paclitaxel (PTX) molecule (Figure 1) is an anti-cancer drug used to treat breast, ovarian, bladder, lung, prostatic, melanoma, and esophageal cancers, as well as Kaposi sarcoma, and other solid tumors [1]. However, it is cytotoxic and interacts in a nonselective manner with healthy and normal cells in addition to cancer cells which consequently causes severe side effects suffered by cancer patients [2].

Targeted delivery using nano-based drug delivery systems can decrease the severe side effects. They can be designed to control the uptake and release of drugs which will improve their bioavailability [3], protect drug molecules [4], create particle sizes smaller than cells that can pass biological barriers and enable targeted delivery [5], and increase durability and biocompatibility in the bloodstream [6]. Several studies have reported the application of nanotechnology for drug delivery systems such as carbon nanotubes [7], graphene sheets [8], liposomes [9], chitosan-based drug carriers [10], carrier proteins, and many more [11,12,13]. One of the challenges in delivering anti-cancer drugs is their low water solubility which is addressed by modifying hydrophobic carriers with hydrophilic attachment or surface-functionalization [11,12]. However, some NDDS, such as carbon-based ones, reportedly affect human biological functions due to their interactions with the cells [14,15,16] and reportedly persist in the human body system for several weeks thus potentially causing long-term toxicity [17,18,19]. In addition, the liposomal formulations which are available in the market limitedly improved the overall survival of treated patients despite improvements in the pharmacokinetic properties of free anti-cancer drugs [20,21]. Therefore, further development and resourcing of nano-based drug delivery systems are needed to reduce cytotoxicity and maximize the therapeutic efficacy of the drugs.

**Figure 2:**
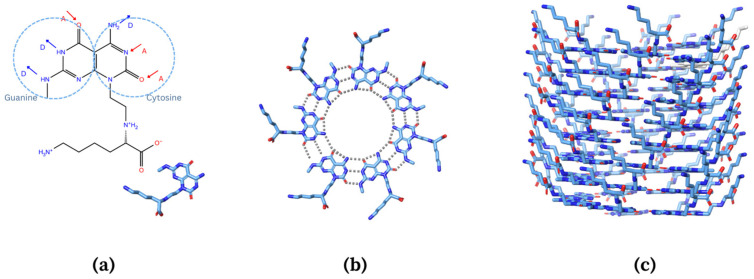
Schematic pathway of the self-assembly of rosette nanotube from (**a**) hydrophobic base unit that possesses Watson–Crick donor–donor–acceptor (DDA) H-bond array of guanine and acceptor–acceptor–donor (AAD) of cytosine to (**b**) formation of rosette ring, and (**c**) stable helix.

The rosette nanotube (RNT), with self-assembling, biocompatible, amphiphilic, and low toxicity properties, has potential as a drug vehicle for PTX [22,23,24,25]. The amphiphilic property of the RNT can help in the loading, delivery, and water solubility of hydrophobic drugs. RNT is more biocompatible than most drug vehicles developed due to its building blocks being a fundamental component of DNA. RNTs are composed of self-assembled supramolecular structures where their basic building blocks possess the Watson–Crick donor–donor–acceptor H-bond array of guanine (G) and the acceptor–acceptor–donor of cytosine (C) (Figure 2a) [23,24,25]. The G^C motifs will undergo self-assembly, then will multiply and form into a rosette ring via hydrogen bonds (Figure 2b). The rosette rings will then form a stable helix and hollow structure (Figure 2c) accordingly due to the dispersion forces, hydrophobic interactions, and π–π stacking interactions [22,24,26]. A notable feature of RNT is that its surface is highly tunable and exceptionally tolerant to a wide range of functional groups, which can improve its solubility and bioavailability [24]. The tubular structure and hollow channels of RNT form a hydrophobic inner channel into which hydrophobic drugs can be incorporated. The surface can be functionalized to enhance targeted delivery [22,24]. With the abovementioned properties, it is hypothesized that RNTs can deliver PTX to the target protein.

**Figure 3:**
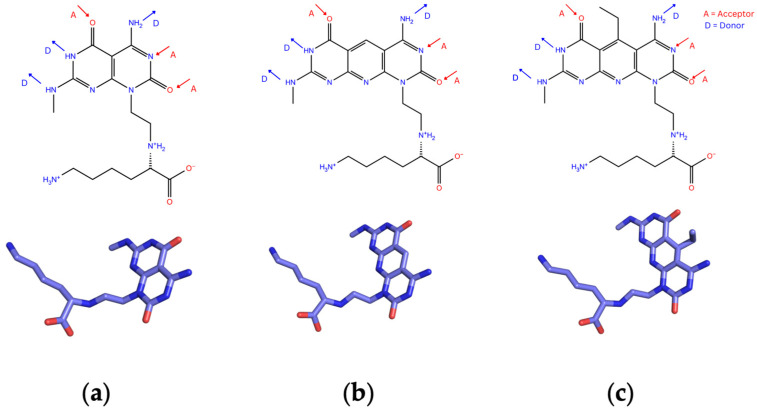
G^C motifs of each type of RNT; (**a**) K1, (**b**)xK1, and (**c**) iEt-xK1 depicting DDA and AAD Hbond arrays [24,26].

Three types of RNTs, namely K1, xK1, and iEt-xK1, were considered in this study. These types vary in the structure of their self-assembling modules, also known as G^C motifs [24,26]. Figure 3a shows the molecular form of the G^C motif for K1 which is in bicyclic form functionalized with lysine to improve its biocompatibility [24]. xK1, on the other hand, is an extended version of K1 where its G^C motif is in tricyclic form (Figure 3b) which is designed specifically to increase the inner diameter of the RNT [24]. iEt-xK1′s G^C motif is similar to xK1′s but with an attached ethyl group in the center ring as shown in Figure 3c [26]. The addition of ethyl group is for the enhancement of the bond between a nonpolar molecule and the inner channel of the RNT.

This study sought to investigate the molecular behavior of PTX against each type of RNT; K1, xK1, and iEt-xK1, to elucidate loading mechanisms, and to determine which among them can be a good drug vehicle candidate for PTX molecules. This will be relevant for the design of the RNT as a drug vehicle for paclitaxel (PTX). We used molecular dynamics (MD) aided with MMPBSA analysis to conduct our study. A limited number of in silico studies have reported the loading of PTX [12]. To the best of our knowledge, no study has tackled the loading of PTX into RNT as the drug vehicle. MD trajectories will provide us insights into the stability, radial distribution of the atoms, and molecular interactions between the PTX and RNT, which will be very relevant to understanding the response of the drug delivery system to the designed conditions and will be fundamental in achieving an efficient and safe formulation of a drug delivery system.

## 2. Results

### 2.1. Molecular Behavior and Stability of Each Type of RNT with PTX Molecule

**Figure 4:**
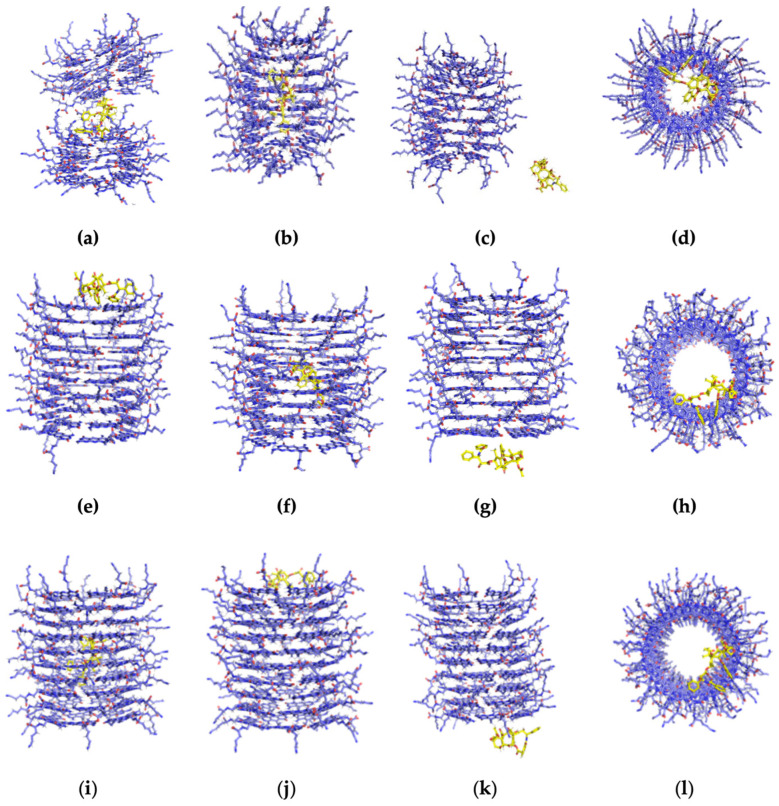
The MD behavior of PTX around the K1 after placing it at different locations at 310 K. The figures shown are (**a**) the horizontal repulsion of K1 with released PTX in the middle, (**b**) the encapsulated PTX in a vertical position (perpendicular to the inner channel of K1), (**c**) the PTX after it was initially placed on outer surfaces of K1, and (**d**) the bottom view of the PTX that has remained on the lower end of the K1, (**e**) the PTX moved upwards from the inner channel of xK1 and remained there, (**f**) the encapsulated PTX by xK1 in an inclined vertical position, (**g**) the PTX approached and remained on the lower end after it was initially placed on the outer surface of xK1, and (**h**) the top view of the PTX that has remained on the upper end of the xK1, (**i**) the encapsulated PTX in a vertical position inside the channel of iEt-xK1, (**j**) the PTX moved upwards the iEt-xK1 and remained there, (**k**) the PTX approached and remained on the lower end after it was initially placed on the outer surfaces of iEt-xK1, and (**l**) the bottom view of the PTX that has remained on the lower end of the iEt-xK1.

Each complex system is comprised of one type of RNT (K1, xK1, iEt-xK1) and one PTX molecule. PTX molecules were placed in different locations against the RNT before they were subjected to MD simulations as the initial states of each complex system. These configurations are (1) in the inner channel parallel to the x-axis (or horizontal) along the length of RNT labelled as the inner channel—horizontal (Figure S2a), (2) in the inner channel parallel to the y-axis (or vertical) along the length of RNT labelled as the inner channel—vertical (Figure S2b), (3) on the side from the outer surfaces of RNT labelled as outer surfaces (Figure S2c), (4) on the upper end surface of the RNT (Figure S2d), and (5) the lower end surface of the RNT (Figure S2e). These configurations are applicable to all types of RNT and were chosen to stage a simulation that will determine the behavior and most stable configuration of PTX against RNT, and explore any possible loading mechanisms. Figure 4 shows the snapshots of one frame for each complex system from the MD trajectories.

A split in the middle of K1 after MD simulation (Video S1) was observed when the PTX was initially placed horizontally in the inner channel as depicted in Figure 4a. This is due to the narrow channel of K1 where the atoms of PTX overlap with atoms of K1 which subsequently caused a crowding effect resulting in steric repulsion. On the contrary, the PTX remained encapsulated when it was initially placed vertically in the inner channel of K1 as shown in Figure 4b (Video S2). This implies that the most probable configuration of PTX inside the channel is in a vertical manner. As to the PTX that was initially placed on the outer surfaces of K1, MD trajectories (Video S3) revealed that it did not interact with the K1 and was unsteady around the system as depicted in Figure 4c. On the other hand, the PTX that was initially placed on the upper and lower end remained in their respective places throughout the simulations (Figure 4d). Similar instances occurred for PTX placed on the upper and lower ends of xK1 and iEt-xK1 (Figure 4h,i).

MD trajectories (Video S4) revealed that PTX gradually moved towards the upper end rosette ring and remained there after initially placing it horizontally in the inner channel of xK1 as shown in Figure 4e. Conversely, the PTX molecule that was initially placed vertically in the inner channel of xK1 remained inside in an inclined vertical configuration shown in Figure 4f (Video S5). This implies that the PTX steadily remains entrapped once it reaches its stable state in the inner channel which is likely in an inclined vertical configuration against the length of the inner channel of xK1. Meanwhile, the PTX shortly ended on the lower end after it was placed on one side from the outer surfaces of xK1 (Figure 4g) as revealed from the MD trajectories (Video S6). 

The opposite behavior was observed for iEt-xK1 with PTX in comparison with the xK1 with PTX complex system. PTX shifted from a horizontal to a vertical position and remained inside the channel of iEt-xK1 shown in Figure 4i (Video S7). Conversely, the PTX moved upwards from a vertical position inside the channel and remained in the upper end as shown in Figure 4j (Video S8). Meanwhile, the PTX eventually attached to the lower end of iEt-xK1 after being placed on the outer surfaces in the last few nanoseconds (Figure 4k). 

**Figure 5:**
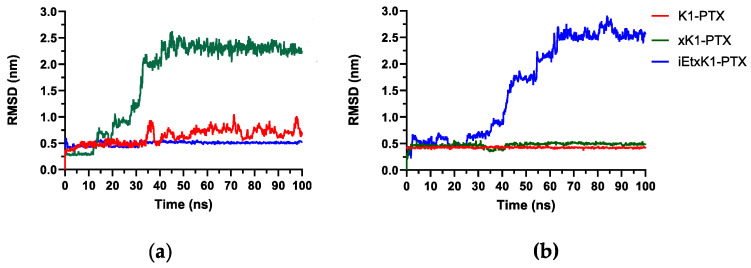
RMSD when PTX was initially placed (**a**) horizontally and (**b**) vertically in the inner channels of each type of RNT as labeled.

Root mean square deviation (RMSD) analysis depicts the stability of the complex system in the designed conditions. It quantifies the conformational changes from the initial state until the final state. The horizontal axis represents the simulation time while the vertical axis represents the RMSD value. Stabilization is deduced upon equilibration of the RMSD plot. RMSD analysis from Figure 5a describes the complex systems wherein PTX was initially placed horizontally in the inner channels of K1, xK1, and iEt-xK1, respectively. RMSD of K1 with PTX (red) revealed fluctuations from the point when steric repulsion occurred (from 35 ns). Stabilization of the complex system can still be inferred despite fluctuations due to small intervals (<0.5 nm). This suggests that another configuration of the PTX with K1. xK1 with a PTX (green) complex system only reaches stabilization after 45 ns when the PTX moves from the inner channel towards the upper end as depicted in Figure 4e. Meanwhile, iEt-xK1 with PTX (blue) in the inner channel is stabilized throughout the simulation. This is when the PTX shifts immediately from a horizontal to a vertical position and remains in the inner channel (Figure 4i). 

Figure 5b describes the complex systems wherein PTX was initially placed vertically in the inner channels of each type of RNT. It shows that K1 (red) and xK1 (green) remained stable throughout the simulation while iEt-xK1 (blue) showed a gradual rise of peaks. K1 and xK1 with the PTX vertically inside their channels exhibit stability because PTX has remained entrapped. On the other hand, the PTX, when initially placed vertically in the inner channel of iEt-xK1, moved towards the upper end resulting to an ascending plot of the RMSD but eventually stabilized around 65 ns. The RMSD results agree with the observed behavior from the MD trajectories. Based on the MD and RMSD results, it shows that PTX vertically inside the channel of each type of RNT is the most probable configuration for encapsulation.

**Figure 6:**
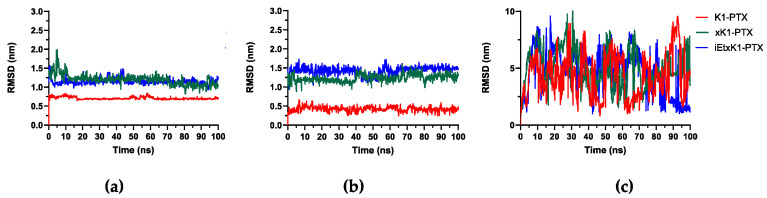
RMSD when PTX was initially placed parallel (**a**) on the upper, (**b**) lower end, and (**c**) on the outer surfaces of each type of RNT as labeled.

Figure 6a,b revealed stabilized complex systems throughout their simulations. Small fluctuations (<1 nm) are observed but they still suggest stability due to small intervals. The described systems are when the PTX remains in its respective places after initially placing it at the upper and lower ends of each type of RNT. Figure 6c describes the RMSD of the complex system wherein PTX was initially placed on the outer surfaces of each type of RNT. It reveals the instability of the systems. PTX eventually ended to the lower ends of xK1 and iEt-xK1 but only occurred on their last 10 ns (Videos S6 and S9), hence still the instability exhibited from their RMSD plots. Figure 6 and the MD results implies that PTX will most likely attach on either end of each type of RNT after they are already self-assembled.

**Figure 7:**
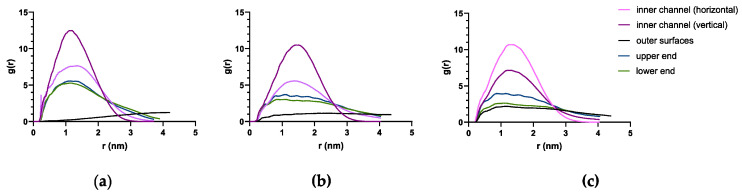
RDF of PTX with (**a**) K1, (**b**) xK1, and (**c**) iEt-xK1 at different locations, respectively.

Radial distribution function (RDF) quantifies the distribution of the PTX at a distance to the RNT. Since the complex system is comprised of only one PTX molecule then the RDF simply describes the occurrence of the PTX at a certain distance to each RNT. A peak at a closer distance (0.5 nm < r < 1.5 nm) is an indicative of PTX being frequent near or within the inner channel. RDF analysis (Figure 7) revealed the sharpest peak (g(r) > 10) and localization at a closer distance to each type of RNT when the PTX remains in the inner channels of each type of RNT. It is followed by a peak wherein PTX remains on either end of each RNT around g(r) of 3–7. Meanwhile, the localization when PTX remains in the middle of the divided K1 (Figure 7a, bright pink) showed close peaks (5 < g(r) < 8) purporting to when the PTX eventually remains on the upper ends of xK1 (Figure 7b, bright pink) and iEt-xK1 (Figure 7c, deep purple). RDF when the PTX was initially placed on the outer surfaces of K1 revealed a linear plot because the PTX did not remain in one place throughout the simulation (Figure 7a, black). Conversely, the RDF for the PTX that was initially placed on the outer surfaces of xK1 and iEt-xK1 showed a slight visible peak (g(r) < 2) in contrast to K1. This was when the PTX occasionally got closer to the lower ends of xK1 and iEt-xK1, explaining the visible peaks. The peaks are more frequent at g(r) > 5 which entails that PTX will likely attach at the ends of the self-assembled RNTs.

### 2.2. Detailed Structural Interaction Analysis between PTX and Each Type of RNT

**Figure 8:**
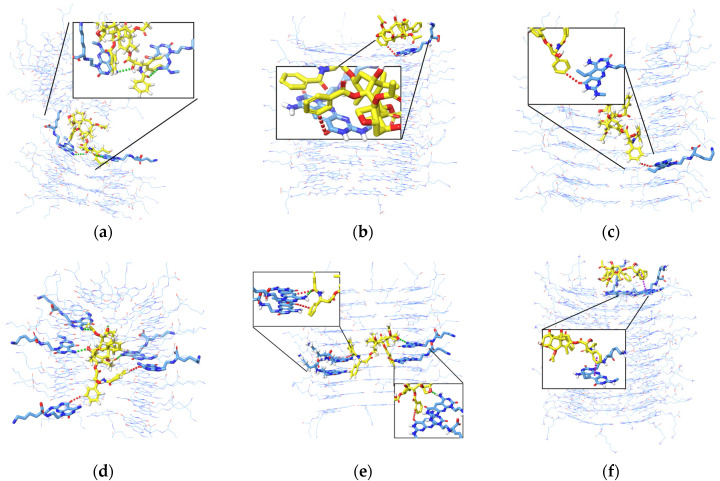
Interaction analysis of the PTX after initially being placed (**a**–**c**) horizontally and (**d**–**f**) vertically in the inner channels of K1, xK1, and iEt-xK1 RNT, respectively, depicting Hydrogen bonding (green dashed lines), aromatic Hydrogen bonding (red dashed lines), and π–π stacking interactions (purple dashed lines). Some G^C motifs were hidden to emphasize the interaction contacts between the PTX and each type of RNT.

Figure 8 shows the interaction analysis for when the PTX was initially placed horizontally and vertically in the inner channels. The frequent interactions that occur from different systems are hydrogen bonds between two H atoms and or H atoms from two aromatic rings. Hydrogen bonds are more evident when PTX is in the divided K1 (Figure 8a) and when PTX is in the inner channels of K1 and xK1 (Figure 8d,e). PTX has only one aromatic H bond with iEt-xK1 when it remains in the inner channel as depicted in Figure 8c. This implies that PTX has a weak hydrogen bond with the iEt-xK1 and the interactions that will most likely occur between the two are interactions among aromatic rings. The instances when the PTX moved and remained on the upper ends of the xK1 and iEt-xK1 have interactions involving aromatic rings such as aromatic hydrogen bonds and π–π stacking as shown in Figure 8b,f. The main interaction that held the PTX specifically in the inner channels of K1 and xK1 is hydrogen bonding. 

**Figure 9:**
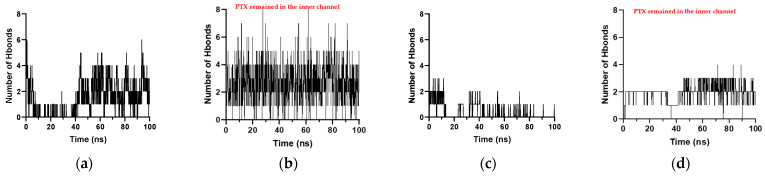
Number of hydrogen bonds against time when PTX was initially placed (**a**) horizontally and (**b**) vertically in the inner channels of the K1, and (**c**) horizontally and (**d**) vertically in the xK1.

Figure 9 shows the hydrogen bond counts of PTX with each type of RNT throughout the simulation. The horizontal axis represents the simulation time while the vertical axis represents the number of hydrogen bonds. The number of hydrogen bonds that occurred for K1 and xK1 when the PTX was initially placed horizontally and vertically in their inner channels is higher and more frequent than when PTX was placed in the inner channel of iEt-xK1. The higher number of hydrogen bond counts is indicative of a strong and stable bond among the interacting molecules. The hydrogen bond counts of PTX with iEt-xK1 throughout the simulation are significantly lower (Figure S1) than with the K1 and xK1 which is indicative of a weaker hydrogen bond between the PTX and iEt-xK1. The introduction of ethyl groups in the G^C motif can be the reason for the weakened hydrogen bond. Meanwhile, hydrogen bonding is stronger when the PTX remains in the inner channels of K1 and xK1 (Figure 9b,d) than when it was in the middle of split K1 (Figure 9a) and when it moved towards the upper end of xK1 (Figure 9c), respectively. This agrees with the observed interactions depicted in Figure 8 where the H bonds are more evident when PTX remains in the inner channels of K1 and xK1. This entails that encapsulation of the PTX by K1 and xK1 can be strengthened by the contribution of hydrogen bonding.

**Figure 10:**
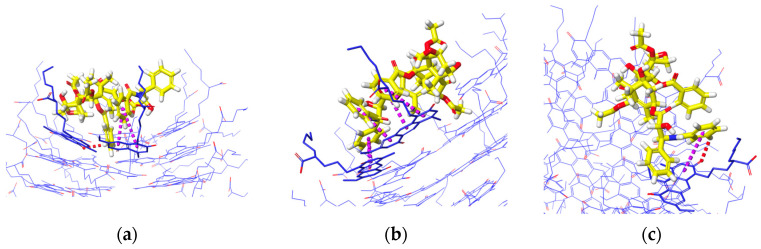
Interaction analysis of PTX when it was initially placed parallel on the upper end of (**a**) K1, (**b**) xK1, and (**c**) iEt-xK1. The usual interaction involved when PTX remains on the upper end is π–π stacking (purple dashed lines).

The interaction analysis from Figure 10 describes the interactions involved when PTX remains on the upper ends of (a) K1, (b) xK1, and (c) iEt-xK1, respectively. The key interactions when PTX remained on the upper ends of each RNT are π–π stacking. It is observed that the π–π stacking interaction when PTX is on the upper ends exhibits a T-shaped π–π stacking orientation wherein the aromatic ring from the PTX approaches one aromatic ring at an angle from the G^C motif.

**Figure 11:**
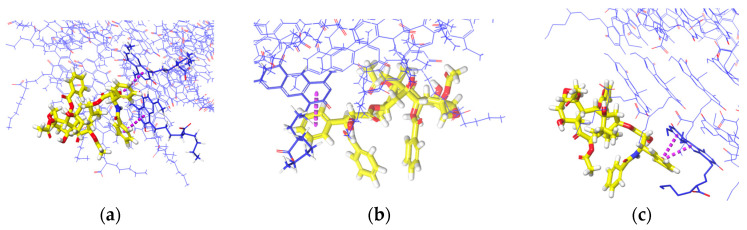
Interactions of PTX when it was initially placed parallel on the lower end of (**a**) K1, (**b**) xK1, and (**c**) iEt-xK1. The usual interactions involved when PTX remains on the lower end is π–π stacking (purple dashed line).

Similarly, the key interactions involved when the PTX remained at the lower ends of K1, xK1, and iEt xK1 type of RNT are mainly π–π stacking as shown in Figure 11a–c, respectively. The π–π stacking interactions when PTX is on the lower ends exhibit a displaced parallel π–π stacking orientation. Displaced parallel π–π stacking is when the PTX aromatic ring is not directly aligned but is slightly parallel from the G^C motif aromatic ring. Generally, parallel π–π stacking is often considered to be more stable than the T-shaped π–π stacking orientation [27]. Both orientations of π–π stacking can contribute to the stabilization of the system but the parallel orientation of π–π stacking allows maximum π-orbital overlap. This results in a larger surface area of interaction and stronger attractive forces between π electron clouds. On the other hand, the T-shaped stacking orientation may vary in distance and orientation of rings which leads to lesser extensive interactions. Thus, PTX can have a stronger bond with the lower ends of each type of RNT than on the upper ends.

**Figure 12:**
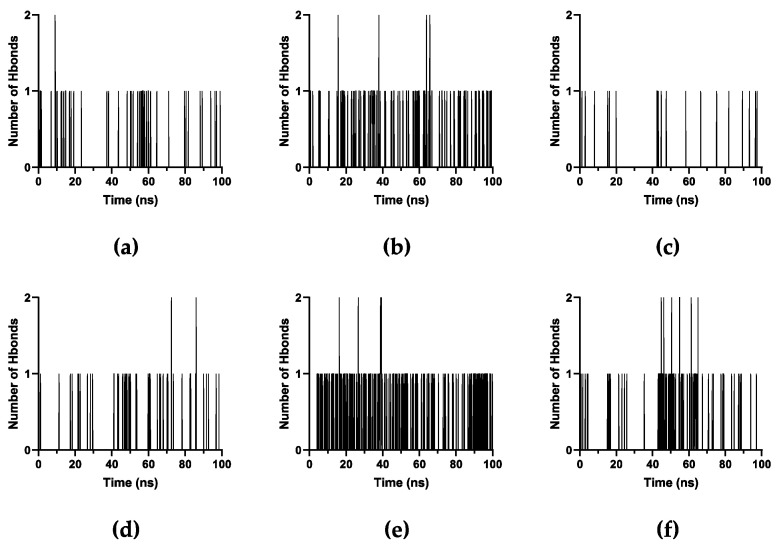
Number of hydrogen bonds against time in picoseconds when PTX was initially placed on the (**a**–**c**) lower and (**d**–**f**) upper ends of K1, xK1, and iEt-xK1, respectively.

The number of hydrogen bonds when PTX is on the upper ends (Figure 12a–c) and lower ends (Figure 12d–f) throughout the simulation is less significant. PTX on lower ends has more frequent hydrogen bonds than when it is on the upper ends, especially for xK1 and iEt-xK1 which makes PTX more stable on the lower ends. Additionally, iEt-xK1 have more hydrogen bond occurring when on either end than when PTX is in the inner channel (Figure S1). Thus, PTX will likely attach on either end rather than being encapsulated by iEt-xK1. Overall, the interactions that will likely occur when PTX is on either end of each RNT are aromatic hydrogen bonds and π–π stacking interactions. That is because of the exposed aromatic rings on either end of each type of RNT.

### 2.3. MMPBSA Analysis 

A single trajectory (STP) protocol approach is applied in the MMPBSA analysis for each system. Thus, the bounded and unbounded states are equal for both receptor (RNT) and ligand (PTX) which makes the total bonded energy (ΔE_Bonded_) equal to zero. The total binding free energy ($∆G_{bind}$) (Equation (1)) is the difference between the receptor’s (G_Rec_) and ligand’s (G_Lig_) binding free energies from the complex system’s (G_Com_). Each binding free energy, G_x_ (Equation (2)) must be the sum of the molecular mechanical energy and the solvation energy of each x, where x refers to the complex, receptor, and ligand, respectively. Thus, $∆G_{bind}$ (Equation (3)) is equal to the sum of the total molecular mechanical energies (ΔE_MM_) and total solvation energy (ΔG_Solv_). Bonded and nonbonded energies combined are the total molecular mechanical energies (ΔE_MM_). Nonbonded energies comprised of van der Waals energy (ΔE_VDW_) and electrostatic energy (ΔE_EE_). The total solvation energy (ΔG_Solv_) is the sum of the polar and nonpolar solvation energies. The polar solvation energy is based on the Poisson Boltzmann (PB) solvent model.

(1) $$∆G_{bind}=[G_{Com}]-[G_{Rec}]-[G_{Lig}]$$

(2) $$\left[G_{x}]=[E_{MM}]+[G_{sol}\right]$$

(3) $$∆G_{bind}={\Delta E}_{MM}+\Delta G_{Solv}$$

The polar solvation energy is positive since both the RNT and the drug molecule are nonpolar. The main contributing energies to the interaction between each RNT and the PTX are nonbonded energies and nonpolar solvation energies. The primary interaction that will pull the drug molecule into the RNT is the hydrophobic interaction among nonpolar molecules as they are solvated in a polar environment (water). The nonbonded interaction will occur once the drug molecule is pulled closer after hydrophobic interactions. Nonbonded interactions mainly depend on the distance of two interacting atoms, thus when the drug molecule is closer to the RNT, van der Waals and electrostatic energy will occur. The behavior or interaction described above is true to all systems. The results from MMPBSA are all based on the last 10 ns frames of the MD trajectories. A more negative value of the binding energy is indicative of a stronger association between the PTX and RNT. On the other hand, a positive value is indicative of a repulsive energy.

One notable binding free energy that differed in the K1-PTX complex is when the PTX was initially placed horizontally in the inner channel, where its binding affinity is significantly higher (weaker energy) than the others at 3384.14 kJ/mol. The main contributor of positive binding affinity is the van der Waals energy as shown in Table 1. This is because of the PTX atoms that are overlapping with some atoms from the inner channel of K1, causing the steric repulsion (Figure 4a). The high magnitude of positive binding affinity can be attributed mostly to the binding affinity between motif molecules of the K1 and less to the interaction between PTX and RNT. Meanwhile, it is observable that the binding free energies (−117.74 and −110.92 kJ/mol) when the PTX was attached to one end (lower or upper) are more negative (stronger) than when the PTX was vertically encapsulated inside the channel of K1 (−45.81 kJ/mol). These results suggest that the most probable configuration of PTX in K1 is horizontally attached on the upper and lower ends with their binding energies stronger.

MMPBSA analysis also revealed that the system with PTX encapsulated in the inner channel of xK1 has the weakest (less negative) binding energy of −53.51 kJ/mol among other xK1 -PTX complexes. But PTX inside the channel of xK1 remained stable given the negative binding energy. However, it was not as strong when it was on either end. Interestingly, the system at which the PTX remained vertically inclined in the inner channel (Figure 4f) showed strong electrostatic energy. This electrostatic energy seemingly contributed the most to the total binding energy of that system. Besides that, hydrogen bonds also contribute to the stabilization of PTX in the inner channel of xK1 as discussed in the previous sections. Meanwhile, significantly higher binding energies are observable when PTX remained on either end. For comparison, PTX has a stronger binding energy of −87.45 kJ/mol the lower end than on the upper end (−69.29 kJ/mol). As recalled on the MD trajectories (Video S6), the PTX eventually ended on the lower end of the xK1 and remained there, especially for the last 10 ns, still showing a strong binding energy of (−78.87 kJ/mol). However, PTX was not captured despite ending up on the lower end. One reason is that a potential energy barrier could occur, specifically on the lower end of the xK1. The difference between the binding energies when the PTX is in the inner channel and when it is on either end is significant, thus an energy barrier is highly possible. An energy barrier is the energy that the drug molecule must overcome to be captured or released in the or from the inner channel of RNT. 

All initial states, except when the PTX was initially placed horizontally in the inner channel, eventually ended up with the PTX on either end of the iEt-xk1. All complex systems at different initial states were stable in their last ten (10) nanoseconds, where their final states were either inside the channel or attached on either end. This resulted in relative binding free energies values (−91.34 to −80.25 kJ/mol) since four (4) out of five (5) systems settled on either end during their final states. The binding energy when PTX remained in the inner channel of the iEt-xK1 is less negative (weaker) at −40.88 kJ/mol than other systems. This indicates that PTX on either end has a stronger bond than when it is in the inner channel of iEt-xK1. Since the PTX molecule comprises three aromatic rings, a π–π interaction could be attributed to the strong association between PTX and the end rosette rings of iEt-xK1 as well as for other types of RNT. Meanwhile, PTX, when it remained inside the channel of iEt-xK1, showed weak electrostatic energy (−2.43 kJ/mol) in contrast to when PTX was in the inner channels of K1 (−49.37 kJ/mol) and xK1 (−147.82 kJ/mol). The addition of the ethyl group could have weakened the electrostatic energy between the PTX and the inner channel of iEt-xK1. Considering the substantial contribution of electrostatic energy in the association between PTX and the inner channels, this could be the reason that iEt-xK1 has the weakest binding energy with PTX in the inner channel. These results from this complex suggest that the most probable conformation of the PTX against iEt-xK1 is horizontally attached at either end.

## 3. Discussion

The structural stability and molecular behavior of the RNT as the drug vehicle for PTX were investigated by molecular dynamics simulations aided with MMPBSA analysis. For each complex system, one (1) PTX molecule was positioned in different locations against each type of RNT. They were positioned in the inner channel both horizontally and vertically, on the outer surface of each RNT, and on both ends of each RNT. Placing the PTX in different locations sought to determine its most stable conformation and to elucidate the most probable loading mechanisms. This present work also sought to determine which of the three RNTs would be most suitable for PTX.

Visual inspection from the MD trajectories revealed steric repulsion of K1 when PTX was placed horizontally in its inner channel. The calculation of MMPBSA energies for the complex after equilibration revealed a value of 3384.14 kJ/mol, indicating steric repulsion. One possible factor for the repulsion is the channel of the K1, which is too tight for PTX when placed horizontally. This made atoms of PTX overlap with some atoms of K1 provoking repulsion of their cloud of atoms. To our surprise, this phenomenon (Figure 4a) eventually stabilized after the PTX remained in the middle and the split RNT did not shift away and remained in their places. This suggests another configuration where PTX can be intercalated between rosette rings, but further computational investigation is necessary. On the contrary, MD trajectories (Video S2) revealed rigid movements of PTX when it vertically remained inside the channel of K1. This is in large part due to the strong hydrogen bonds and favorable binding energy of −45.81 kJ/mol between the PTX and the inner channel of K1. K1 has strong energetic barriers as reported in one study, which could be one of the reasons why PTX remained with restricted movements inside the channel [26]. This implies that the PTX can be entrapped once it has reached its most probable configuration in the inner channel of K1. Otherwise, a steric repulsion may occur. K1 exhibited a strong binding affinity of −117.74 kJ/mol with PTX when it equilibrated towards one end of the RNT. These observations and results imply that the most probable way of loading PTX in the K1 is by attachment on either end. 

Further visual inspection revealed that both xK1 and iEt-xK1 maintained their initial shapes better than K1 throughout the simulation. This implies that xK1 and iEt-xK1 are more stable in physiological conditions. MD trajectories also revealed that PTX eventually moved from the inner channel towards the upper ends of xK1 and iEt-xK1 and remained there. This case could be associated with the energetic barriers for xK1 and iEt-xK1, where it was reported that they do not exhibit strong energy barriers unlike K1 [26]. However, MMPBSA results revealed significant binding energies of −78.87 and −80.25 kJ/mol when PTX was initially placed on the outer surfaces and eventually ended at the lower ends of xK1 (Video S6) and iEt-xK1 (Video S9), respectively. The hindrance of the intake of PTX in the inner channels despite ending up on the lower end could be attributed to the possible energy barrier, particularly on their lower ends. On the other hand, the escape of the PTX from the inner channels toward the upper end is attributed to the weak energy barriers reported in one study [26]. 

The PTX remained inside the channel of xK1 and iEt-xK1 in an inclined vertical position against the length of each RNT (Figure 4f,i) which is similar to the phenomena when PTX remained vertically inside the channel of K1 (Figure 4b). RMSD analysis revealed that such systems remained stable throughout their simulation and their MMPBSA analysis consistently resulted in favorable binding energies of −53.51 to −40.88 kJ/mol. Even though they are less negative (weaker) than when PTX remains on either end of each RNT, their binding energies are still significant, demonstrating a strong association between PTX and the inner channel of each RNT. These observations particularly for xK1 and iEt-xK1 imply that PTX can be captured during the self-assembly process and will tend to move upwards and remain on the upper ends. On the other hand, the PTX from the bulk solution after the stabilization will end up on the lower ends. In general, encapsulation of the PTX during the self-assembly process for all types of RNT is possible after it reaches its most probable state in the inner channel due to the favorable binding energies despite being weaker (more positive) than other configurations. 

Our findings lead to the proposed loading mechanisms of PTX onto each type of RNT as follows (1) the PTX approaches the edge of self-assembled RNT from the bulk solution and will remain steady on either end and (2) the encapsulation of the PTX by xK1 and iEt-xK1 will occur during the self-assembly process until it is stabilized. In regard to the releasing mechanism, cancer cells are inherently in acidic conditions, hence carriers that react with the change in pH are preferable [28,29]. These carriers are expected to break down at reduced pH and simultaneously release the drugs into the target cell. Several studies [3,30,31,32] reported the investigation of releasing anti-cancer drugs via MD simulations with pH-sensitive carriers. Studies reported that cytosine protonates with reduced pH, thus its motif unfolds in an acidic environment [33,34]. This phenomenon suggests that RNT, with base units made up of guanine and cytosine base pairs, is capable of releasing the Paclitaxel in the target cancer cells. It is expected that RNT will destabilize with protonated cytosine in an acidic environment which will disrupt the hydrogen bond network holding the RNT together. Further investigation is needed to verify this. In summary, we succeeded in showing that each type of RNT is stable and has a strong binding affinity with PTX in most configurations under physiological conditions. This demonstrates the suitability of each RNT as a drug vehicle. K1 particularly showed favorable binding energies and stability; however, its inner channel is too narrow to accommodate PTX for encapsulation. On the other hand, xK1 exhibits structural stability and favorable binding energies in physiological conditions both when the PTX remains inside the channel and on either end, whereas iEt-xK1 revealed a lower binding strength with PTX in the inner channel than K1 and xK1. From this, xK1 is revealed to be a good drug vehicle candidate for PTX among other types of RNTs. Nevertheless, each type of RNT showed favorable results, which warrants further experimental studies.

## 4. Materials and Methods

### 4.1. Modeling

Three different types of RNT were used here: K1, xK1, and iEt-xK1 [24,26]. Each motif molecule was prepared and built using Schrödinger Maestro 2021-1 [35] and was optimized using OPLSAA forcefields. The built motif was multiplied into a rosette ring and stacked in a tubular manner. The modeling and parameters of the RNT were adapted from several studies [23,24,26] described and tabulated in Figure S3 and Table S1, respectively. The molecular structure of the PTX was obtained from PubChem [36]. The topology parameters of each motif molecule and PTX were generated from LigParGen [37]. Each PTX was placed in five (5) different locations against each type of RNT. These are (1) horizontally in the inner channel (inner channel horizontal (2) vertically in the inner channel (inner channel vertical), (3) on the outer surfaces, (4) on the upper end, and (5) on the lower end shown in Figure S2.

### 4.2. Molecular Dynamics

All molecular dynamics simulations were performed using GROMACS 2021.4 [38]. The RNT with the drug molecule complex systems were prepared by minimizing, solvating, and adding ions to each system accordingly with OPLSAA forcefield parameters. The minimization model is performed using the steepest descent, while the water model used for solvating is TIP3P and the ion concentration added is 0.15 M NaCl. The equilibration is conducted through two phases. The first phase is performed using an NVT ensemble, also termed isothermal isochoric, or canonical. The NVT ensemble is used to stabilize the temperature. It is followed by an NPT ensemble to stabilize the pressure. The simulation time was set to 100 ns, while the temperature was set to 37 °C to conform with the physiological conditions. The force field used for this MD simulation is OPLSAA as well. MD trajectories were subjected to root mean square deviation (RMSD) analysis and radial distribution function (RDF) analysis. RMSD and RDF analyses were executed to confirm or validate the molecular behavior observed from the MD trajectories. The RMSD analysis was based on the stability of the drug molecule with respect to the RNT. RDF analysis establishes the radial distribution of certain atoms at a distance from the reference atoms. The described atoms are from the PTX molecule, while the reference atoms are from the RNT. Thus, the RDF plot reveals the PTX molecule’s distribution at a distance from the RNT. Each complex system is comprised of one kind of RNT (K1, xK1, iEt-xK1) with a PTX molecule placed in different locations with a total of fifteen (15) complex systems.

### 4.3. MMPBSA Analysis

MMPBSA analysis was used to estimate the binding free energies of the drug molecules against each type of RNT. The gmx_mmpbsa tool [39] was used for this analysis which uses the obtained MD trajectories from GROMACS. This tool utilizes AmberTools [40] along with GROMACS. The calculation is based on AMBER’s MMPBSA.py [41], thus the topologies for OPLSAA ff in GROMACS format were converted to AMBER format. This conversion is performed simultaneously using parmed [42]. The contemporary approach to calculating the nonpolar solvation energy is not applicable because it results in unrealistic solvation energies for each complex system. The obtained results were tabulated according to each system.

## Figures and Tables

**Table 1:** MMPBSA analysis of each type of RNT with PTX complex systems in different locations.

PTX Locations	ΔG_Bind_ (kJ/mol)	ΔE_VDW_ (kJ/mol)	ΔE_EEL_ (kJ/mol)	ΔG_PB_ (kJ/mol)	ΔG_NP_ (kJ/mol)	ΔE_MM_ (kJ/mol)	ΔG_Solv_ (kJ/mol)
K1-RNT with PTX							
Inner Channel (horizontal) ^1^	3384.14	3239.34	−118.74	291.29	−27.74	3120.64	263.51
Inner Channel (vertical) ^2^	−45.81	−214.35	−49.37	240.29	−22.34	−263.76	217.94
Outer surfaces	−3.93	−0.88	−4.94	99.87	−0.17	−5.82	9.75
Upper end	−110.92	−203.01	−58.74	171.67	−20.84	−261.75	150.83
Lower end	−117.74	−198.36	−31.46	66.94	−17.82	−166.90	49.16
xK1-RNT with PTX							
Inner Channel (horizontal) ^3^	−101.50	−173.64	16.23	71.76	−15.82	−157.44	55.94
Inner Channel (vertical) ^2^	−53.51	−98.74	−147.82	205.94	−12.84	−246.60	193.09
Outer surfaces ^4^	−78.87	−139.41	62.47	10.25	−12.18	−76.94	−1.88
Upper end	−69.29	−120.00	21.80	41.09	−12.18	−98.20	28.91
Lower End	−87.45	−131.00	−67.78	123.60	−12.26	−198.78	111.34
iET-xK1-RNT with PTX							
Inner Channel (horizontal) ^2^	−40.88	−87.95	−20.75	79.91	−12.13	−108.70	67.78
Inner Channel (vertical) ^3^	−91.34	−170.21	−2.43	97.95	−16.65	−172.63	81.30
Outer surfaces ^4^	−80.25	−122.93	−36.86	90.92	−11.42	−159.75	79.50
Upper end	−71.71	−117.78	−54.68	112.63	−11.88	−172.42	100.75
Lower end	−87.45	−131.00	−67.78	123.60	−12.26	−198.78	111.34

^1^ MMPBSA results when K1 split in half with PTX remained in between ^2^ MMPBSA results when PTX remained inside the channel of K1 ^3^ MMPBSA results when PTX flowed out to one end of the RNT ^4^ MMPBSA results when PTX approached the lower end of xK1 and iEt-xK1.

## Data Availability

The data presented in this study are available in the article and the Supplementary Materials.

## Supplementary Materials

The following supporting information can be downloaded at: https://www.mdpi.com/article/10.3390/molecules28237853/s1, Figure S1: Number of hydrogen bonds when PTX was placed initially in the inner channel of iEt-xK1. Figure S2: Locations of PTX molecules on each type of RNT; Figure S3: Description of the parameters of Table S1; Table S1: Optimal parameters of each type of RNT; Video S1: MD trajectories of K1 with PTX (inner channel horizontal); Video S2: MD trajectories of K1 with PTX (inner channel vertical); Video S3: MD trajectories of K1 with PTX (outer surfaces); Video S4: MD trajectories of xK1 with PTX (inner channel horizontal Video S5: MD trajectories of xK1 with PTX (inner channel vertical); Video S6: MD trajectories of xK1 with PTX (outer surfaces); Video S7: MD trajectories of iEt-xK1 with PTX (inner channel horizontal); Video S8: MD trajectories of iEt-xK1 with PTX (inner channel vertical); Video S9: MD trajectories of iEt-xK1 with PTX (outer surfaces).
