# Supplementary material for: In Silico Investigation on the Molecular Behavior and Structural Stability of the Rosette Nanotubes as the Drug Vehicles for Paclitaxel, an Anti-Cancer Drug
Source: Molecules. 2023 Nov 29;28(23):7853. doi: 10.3390/molecules28237853 (PMC10708515; doi:10.3390/molecules28237853)
Supplement: Supplementary file 1 [file molecules-28-07853-s001.zip › Supplementary Information.pdf]

Supplementary Information

Video S1-S9: MD trajectories of each complex system

S.1 to S.3 are the MD trajectories of K1 type of RNT with the PTX, S4 to S6 are the MD trajectories for xK1 type of RNT with the PTX, and S7 to S9 are the MD trajectories for iEt-xK1 type of RNT with the PTX.

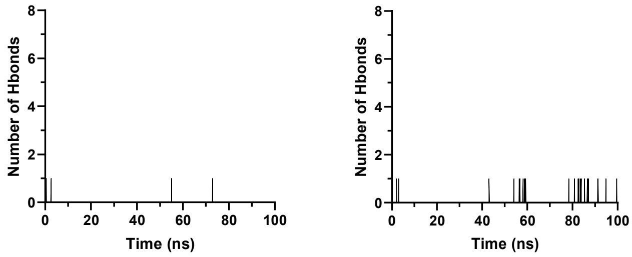

(a) (b)

Figure S1. Number of hydrogen bonds against time when PTX was initially placed (a) horizontally and (b) vertically in the inner channel of iEt-xK1

Figure S2. Locations of PTX molecules on each type of RNT

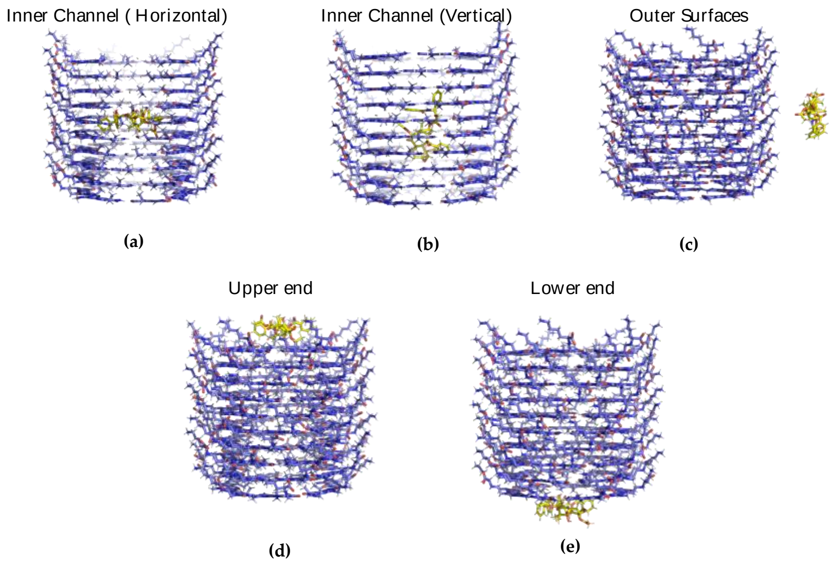

Figure S2. shows the different initial states of the drug molecule and rosette nanotube systems before they underwent MD simulations. These locations are applicable to all kinds of RNT (K1, x1, iEt-xK1) Figure S2a. shows the drug molecule positioned parallel to the x-axis (or horizontal) of

the inner channel of any RNT termed as "Inner channel (horizontal)" Figure S2b termed as " Inner channel (vertical)" is the system where the drug molecule is positioned parallel to the y-axis (or vertical) of the inner channel of any RNT. Figure S2c. termed as "outer surfaces" is the system wherein the drug molecule was initially positioned away from the RNT. Figure S2d. termed as "upper end" is the system at which the drug molecule is placed on the upper end surface of the RNT. Lastly, Figure S2e. termed as "lower end" is the system in which the drug molecule is positioned on the lower end surface of the RNT.

Figure S3. Description of the parameters

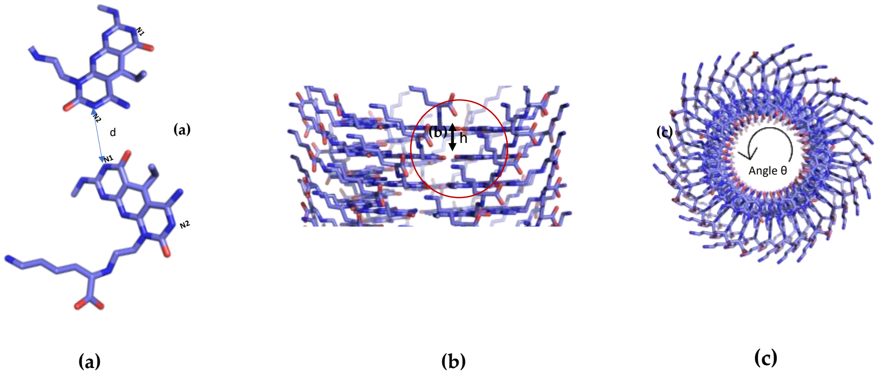

Figure S3. Description of the parameters mentioned in Table S1.

Figure S3a. shows the distance of the N atom (N1) from the guanine of one motif to the N atom (N2) of the cytosine from the other motif. While Figure S3b. shows the height between the two stacked rosette rings whether it may be stacked or helical. Figure S3c. represents the angle of rotation of each rosette ring from the other rosette ring.

Table S1. Optimal parameters of each type of RNT

Type of RNT	d (N1 – N2 distance) Å	H (rosette stacks height) Å	Angle θ	r (helical radius) Å
**K1**	3.0	3.45	30.0	9.0
**xK1**	2.95	3.35	-14.3	-
**iEt-xK1**	2.95	3.35	-14.3	-

The motif for each type of RNT was manually created using the builder tool from Maestro and optimized simultaneously. Each optimized motif molecule was used to form rosette rings. Each rosette ring was stacked for up to 10 rings in a tubular structure [28]. Each type of RNT has corresponding optimal parameters. Optimal parameters are necessary to ensure the stability of the RNT in terms of the fit of the drug molecules inside the channel without blowing up the complex

system. These must be ensured before combining each type of RNT with the drug molecules to form a complex system. These optimal parameters were obtained from running MD simulations. Table S1 shows the optimal parameters for the RNTs to fit the PTX in the inner channel. The K1 is stable in a right-handed helical tubular structure. Thus, its helical radius must be specifically indicated.
